# A Digital Health Intervention (SweetGoals) for Young Adults With Type 1 Diabetes: Protocol for a Factorial Randomized Trial

**DOI:** 10.2196/27109

**Published:** 2021-02-23

**Authors:** Catherine Stanger, Tobias Kowatsch, Haiyi Xie, Inbal Nahum-Shani, Frances Lim-Liberty, Molly Anderson, Prabhakaran Santhanam, Sarah Kaden, Briana Rosenberg

**Affiliations:** 1 Center for Technology and Behavioral Health Geisel School of Medicine Dartmouth College Lebanon, NH United States; 2 Centre for Digital Health Interventions Department of Management, Technology, and Economics ETH Zurich Zurich Switzerland; 3 Centre for Digital Health Interventions Institute of Technology Management University of St Gallen St Gallen Switzerland; 4 Institute for Social Research University of Michigan Ann Arbor, MI United States; 5 Dartmouth-Hitchcock Medical Center Lebanon, NH United States

**Keywords:** type 1 diabetes, mhealth, incentives, health coaching, young adults

## Abstract

**Background:**

Many young adults with type 1 diabetes (T1D) struggle with the complex daily demands of adherence to their medical regimen and fail to achieve target range glycemic control. Few interventions, however, have been developed specifically for this age group.

**Objective:**

In this randomized trial, we will provide a mobile app (SweetGoals) to all participants as a “core” intervention. The app prompts participants to upload data from their diabetes devices weekly to a device-agnostic uploader (Glooko), automatically retrieves uploaded data, assesses daily and weekly self-management goals, and generates feedback messages about goal attainment. Further, the trial will test two unique intervention components: (1) incentives to promote consistent daily adherence to goals, and (2) web health coaching to teach effective problem solving focused on personalized barriers to self-management. We will use a novel digital direct-to-patient recruitment method and intervention delivery model that transcends the clinic.

**Methods:**

A 2x2 factorial randomized trial will be conducted with 300 young adults ages 19-25 with type 1 diabetes and (Hb)A_1c_ ≥ 8.0%. All participants will receive the SweetGoals app that tracks and provides feedback about two adherence targets: (a) daily glucose monitoring; and (b) mealtime behaviors. Participants will be randomized to the factorial combination of incentives and health coaching. The intervention will last 6 months. The primary outcome will be reduction in A_1c_. Secondary outcomes include self-regulation mechanisms in longitudinal mediation models and engagement metrics as a predictor of outcomes. Participants will complete 6- and 12-month follow-up assessments. We hypothesize greater sustained A_1c_ improvements in participants who receive coaching and who receive incentives compared to those who do not receive those components.

**Results:**

Data collection is expected to be complete by February 2025. Analyses of primary and secondary outcomes are expected by December 2025.

**Conclusions:**

Successful completion of these aims will support dissemination and effectiveness studies of this intervention that seeks to improve glycemic control in this high-risk and understudied population of young adults with T1D.

**Trial Registration:**

ClinicalTrials.gov NCT04646473; https://clinicaltrials.gov/ct2/show/NCT04646473

**International Registered Report Identifier (IRRID):**

PRR1-10.2196/27109

## Introduction

### Type 1 Diabetes in Young Adults

The incidence of type 1 diabetes (T1D) is rising [1], and T1D results in significant economic costs in the United States, with yearly medical expenditures estimated at approximately $7 billion with an additional $7 billion in lost wages [2]. T1D also significantly increases mortality, especially among those with above target hemoglobin A_1c_ (HbA_1c_) levels [3]. Young adults are a population at unique risk, with only 14% of young adults aged 18 to 25 years meeting the target HbA_1c_ goal (HbA_1c_≤7%) versus 30% of those over 30 years [4]. One in 4 young adults aged 18 to 35 years already have one or more medical complications related to their T1D, most commonly renal problems reflecting micro- or macroalbuminuria and/or retinopathy [5]. Furthermore, young adulthood is a critical developmental period when adult habits are formed as patients transition from parental involvement with diabetes management to independence in self-management of their T1D [6,7].

Despite the unique clinical needs of patients in this age group, few interventions have been tested for this high-risk population. A 2017 systematic review found 18 intervention studies for young adults with T1D [8]. Across studies, the most common intervention strategy (13/18, 72%) targeted engaging young adults with clinical services, an important goal, but unfortunately one that did not routinely result in improved glycemic control in most trials. Only 67% (12/18) of studies reported HbA_1c_ outcomes and, of these, only 2 were randomized, both showing no impact on HbA_1c_. Since this review, several protocols and intervention development studies have been published [9,10]; several reported the results of uncontrolled studies with none showing impact on HbA_1c_ to date [11-13] and one showing significant effects on HbA_1c_ for continuous glucose monitor (CGM) use versus blood glucose meters [14]. These results highlight a major gap in and need for more rigorous research on effective ways to improve glycemic outcomes among young adults with T1D.

### Intervention Model

**Figure 1:**
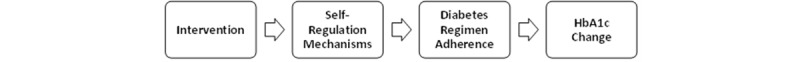
Conceptual model of intervention effects on hemoglobin A_1c_.

The proposed intervention model offers a multipronged self-regulation approach for targeting glycemic control that is tailored for young adults. The goal of the selected intervention components is to improve self-regulatory mechanisms [15] including self-monitoring, goal setting, self-efficacy about diabetes management, and problem-solving skills. These self-regulatory mechanisms promote improved T1D regimen adherence and HbA_1c_ [16-20]. The conceptual model in Figure 1 highlights the role of self-regulation as an intervention target leading to improved outcomes. To target self-regulation among youth with T1D, we developed a multicomponent intervention that includes (1) weekly diabetes device data upload and data review designed to promote healthy self-monitoring and goal-setting habits for diabetes management and provide feedback about goal attainment, (2) web-based human coaching to deliver motivational interviewing exercises and teach a structured problem-solving method, and (3) motivational incentives to enhance adherence.

A series of prior iterative studies developed and tested this intervention approach, including a randomized trial comparing a similar intervention to usual care for adolescents aged 13 to 17 years. Intervention youth had significantly lower HbA_1c_ levels at the end of the 6-month intervention (*d*=.45), and this effect was fully maintained indicating no weakening of the intervention effect at the 12-month follow-up (*d*=.44) [21]. Our new study adapted this earlier intervention to enhance both efficacy and disseminability. The primary modification to increase efficacy involves expanding the goal target from glucose checking to encompass the additional key self-management behavior of carbohydrate counting. Enhancements to promote dissemination (and scalability) include (1) modifying goals to encompass the full spectrum of diabetes devices, (2) automating the goal setting and feedback components via an app, and (3) recruiting young adults via social media. In addition, this study uses a factorial design to test the independent effects of incentives to promote consistent daily adherence to goals and web health coaching to teach effective problem solving focused on personalized barriers to self-management, providing better understanding of the intervention mechanisms.

### Incentives to Promote Self-Management

Incentives may be an effective tool for increasing self-management behavior. Because interventions that support self-monitoring of diabetes management have shown limited effects on HbA_1c_, incentives are designed to enhance the impact of the intervention on HbA_1c_. Consistent with behavior economic theory [22], most daily adherence behaviors necessary to manage T1D do not result in immediate positive experiences. The benefits from consistent, daily adherence accrue over weeks, months, years, and decades of life. The use of immediate incentives for adherence is one way to increase the value of such behaviors in the present, providing an immediate reason to adhere. In the SweetGoals intervention, incentives target improvement in specific self-management behaviors, and such improvement is expected to improve glycemic control. Three studies [23-25] using incentives for glucose checks have shown significant positive effects, as have our prior studies [21,26,27].

### Health Coaching to Improve Self-Regulation

This study will also test the impact of a coaching intervention focused on enhancing motivation and teaching problem-solving skills to promote long-term outcomes. There is evidence that motivational interviewing and instruction in problem-solving skills can improve medical adherence including in T1D [28-35]. Health coaches will teach these skills in brief web-based sessions in the context of device data review focused on actionable self-management targets. The curriculum begins with motivational exercises to guide selection of concerns that become the target of the problem-solving sessions. The health coach also facilitates engagement with self-monitoring and goal-setting habits using the Glooko data visualization platform and the SweetGoals app goal feedback, consistent with the supportive accountability model [36]. Providing such support increases the efficacy of digital interventions [37-42], and delivery by bachelor’s level coaches can be as effective as professional clinicians across diverse clinical targets [43,44]. The supportive accountability model emphasizes the key role that social presence (human coach) plays in setting clear expectations regarding adherence to the steps necessary to achieve a positive outcome (eg, glucose monitoring) and in supporting goal attainment via progress monitoring and feedback [36]. Our pilot results strongly support the long-term sustained efficacy of this coaching approach combined with incentives [21], but those results cannot inform the need for both of these distinct interventions. This new study will replicate those earlier results in a novel population of young adults and test the separate impact of incentives, coaching, and their interaction.

## Methods

### Participants

We will enroll 300 young adults with T1D, aged 19 to 25 years (target 50% female) who have HbA_1c_≥8%. Young adults must use a Glooko compatible glucometer or CGM and may use either multiple daily injections (MDI) or continuous subcutaneous insulin infusion (CSII). See Textbox 1 for detailed inclusion and exclusion criteria.

**Inclusion and exclusion criteria for the SweetGoals study.**

Inclusion criteria:

- Diagnosis of type 1 diabetes for longer than 18 months
- HbA_1c_ ≥8.0%
- Report a visit with physician managing type 1 diabetes within the previous 6 months
- Participants must use a glucometer or continuous glucose monitor compatible with Glooko

Exclusion criteria:

- Pregnancy or breastfeeding
- Severe medical illness that would preclude participation (eg, cystic fibrosis, developmental disability, severe cognitive impairment)
- Psychiatric illness that would preclude participation
- Diabetes diagnosis other than type 1 diabetes (type 2 diabetes, maturity onset diabetes of the young)
- Use of any medications known to impact glycemic control (oral or injectable corticosteroids, beta-blockers, antipsychotic medications such as risperidone)
- History of known hemoglobinopathy, anemia, or transfusion (which could alter the validity of HbA_1c_ measurement)
- Already being engaged in a psychological intervention targeting diabetes adherence

### Recruitment

**Figure 2:**
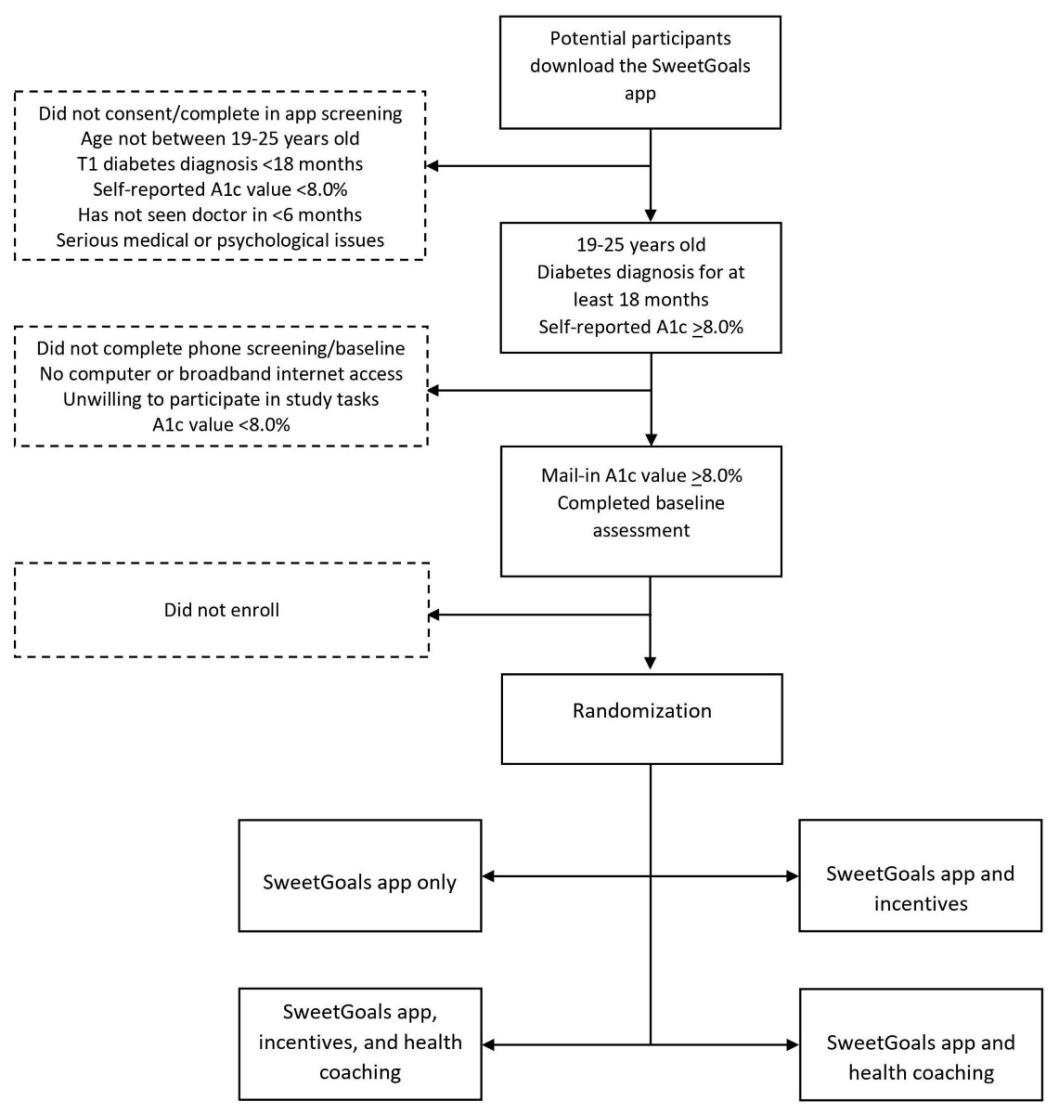
Screening, recruitment, and randomization process.

Participants will be recruited using Facebook, Instagram, and Google ads. Best practices for ethical recruitment using social media platforms will be followed [45,46]. Breaks will occur between ad runs to reduce ad fatigue. The ads will prompt potential participants to download the SweetGoals app. Once they confirm that they are in the target age range, they will complete the study consent in the app. Consenting participants will complete a brief survey via the app to confirm eligibility. Research staff will follow up to complete the screening process, confirming device compatibility with Glooko and arranging for completion of the HbA_1c_ test via postal mail. Once eligibility is confirmed (mail-in HbA_1c_≥8%), staff will help participants create their Glooko accounts and install the Glooko uploader and Glooko smartphone app via phone or a web video chat. Staff will then complete the randomization as described below. Figure 2 displays the screening, recruitment, and randomization process.

### Procedure

A fully powered 2×2 randomized factorial experiment (see Table 1) will be used to evaluate specific intervention components in terms of HbA_1c_ outcomes. Factorial designs are a highly efficient experimental approach for answering questions about the utility of multiple intervention components and their combinations. Data from these designs can be used to test the main effect of each intervention component as well as their interaction [47,48]. All participants will receive the SweetGoals app. Those assigned to the incentive group will receive incentives for meeting glucose monitoring and mealtime targets. Those assigned to the coaching group will receive web coaching in problem-solving skills focused on achieving better self-management and glycemic control. If assigned to incentives, participants will receive incentives weekly for 3 months, with gradually fading frequency over the next 3 months. If assigned to coaching, participants will meet with the coach weekly via web video for 3 months, with the frequency of those meetings also fading gradually over the next 3 months. The intervention period lasts for 6 months. Follow-up assessments will be completed at 6 months and 12 months after baseline.

**Table 1:** Experimental conditions of the 2×2 factorial design.

Experimental condition	SweetGoals app	Incentives	Web coaching
1	Yes	No	No
2	Yes	No	Yes
3	Yes	Yes	No
4	Yes	Yes	Yes

Each component will take on 2 levels: yes or no. Note that the factorial design in Table 1 is not a 4-arm trial with each condition compared with a control or to each other. Instead, our interest is in tests of standard analysis of variance main effects and interactions [49]. These involve comparison of outcome means across multiple experimental conditions. For example, the main effect of the incentives component will be tested by comparing the mean of the outcome variable across the 2 conditions in which incentives will be delivered (ie, those in conditions 3 and 4; n=150 before attrition) versus the 2 conditions in which incentives will not be delivered (ie, those in conditions 1 and 2; n=150 before attrition). Hence, this main effect will be tested by comparing half of the sample (those offered incentives) versus the other half (those not offered incentives) in terms of the primary outcome. In this factorial experiment, each effect (including the interaction between the 2 components) will be estimated based on data from all 4 conditions (ie, the full sample) [48,50,51].

**Figure 3:**
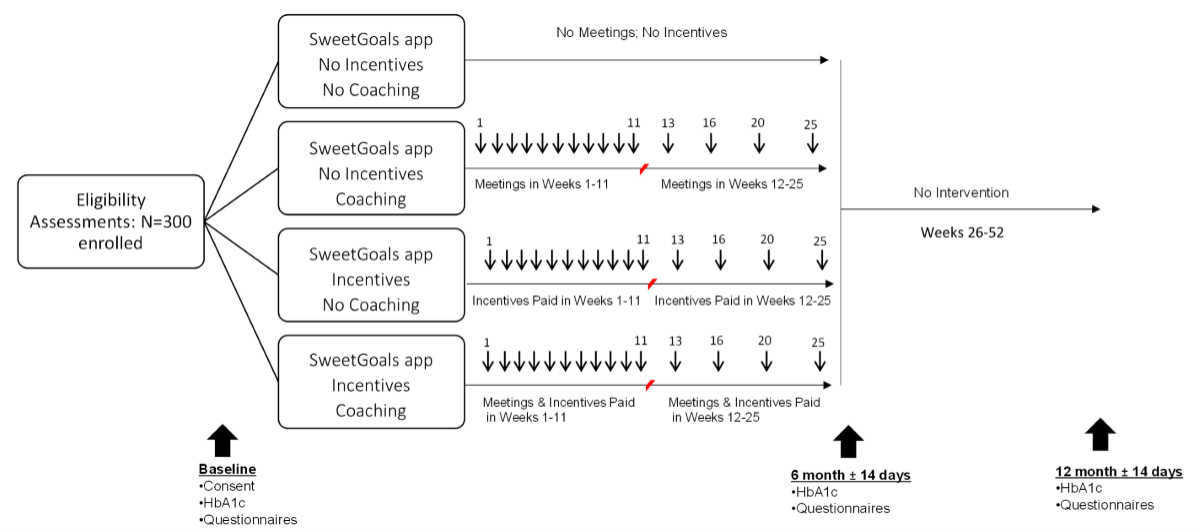
Study design.

Figure 3 shows the study design. An online minimization program (MinimPy) will be used to assign participants [52]. Minimization assures similarity across intervention groups on multiple key covariates [52]. Differences between conditions will be minimized on gender, age, ethnicity (minority vs White), CSII versus MDI, CGM use, and HbA_1c_. Follow-ups will occur at 6 months and 12 months. Compensation will be $25 for the baseline assessment, $50 for each follow-up assessment, and a supplement of $50 for completing both follow-ups. This study was approved by the Committee for the Protection of Human Subjects at Dartmouth College and is registered at ClinicalTrials.gov [NCT04646473].

### SweetGoals App Core Intervention

**Figure 4:**
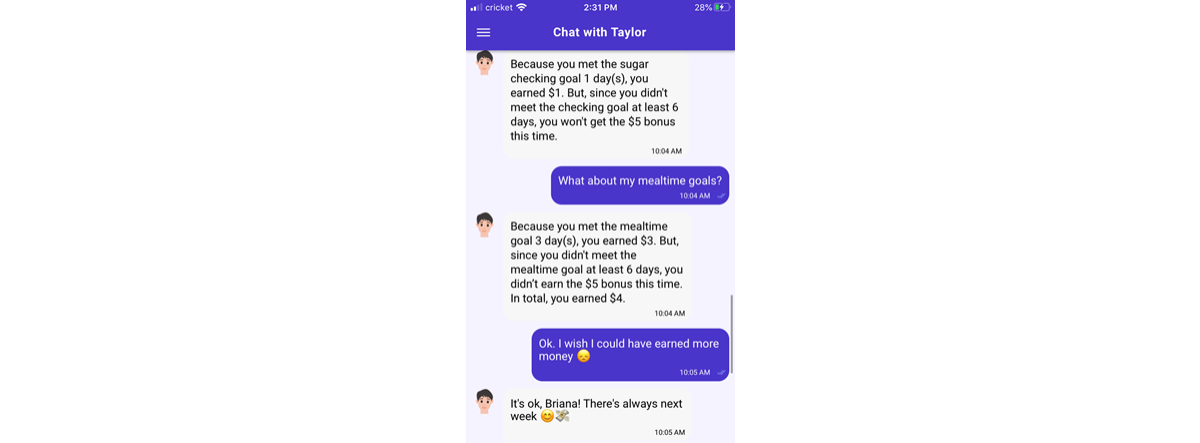
SweetGoals dialogue with digital coach Taylor.

All participants will receive SweetGoals, an app that sends messages about self-management goals, goal adherence, and encouragement about adherence in the upcoming week. Participants will receive messages on Sunday reminding them to upload their data and feedback detailing their goal achievement on Monday. They will also receive educational materials in a second message most weeks (eg, links to information about self-management of T1D). The app is programmed in MobileCoach [53-55], an open-source app platform that has the functionality to integrate our goal-tracking algorithms and provide messages that prompt device uploads and provide automated feedback. MobileCoach sends messages written by the research team in the style of Facebook Messenger or WhatsApp from a digital coach who communicates with the app user to provide scripted feedback about goals as shown in Figure 4. Participants select from a random sample of 4 coaches offered from a bank of 12 coaches of diverse gender and race/ethnicity. The participant (Briana in Figure 4) can answer the digital coach (Taylor in Figure 4) using predefined responses.

### Daily and Weekly Self-Management Goals

The SweetGoals app will automatically download the participant’s device data each Sunday at midnight using the Glooko application programming interface. The app will use the device data to provide feedback each Monday about goals that target glucose monitoring and mealtime behaviors tailored by device as shown in Table 2. The glucose monitoring goal for participants who use a glucometer only (no CGM) is ≥5 checks, each ≥2 hours from another check. Additional checks—regardless of spacing—are encouraged based on clinical needs (eg, hypoglycemia). This same criterion was used successfully in prior studies [21,26,27]. For participants who use CGM, we conservatively define adequate daily CGM wear time as 80% of expected values each day. We selected this daily glucose monitoring goal to provide clinically meaningful data to the participant but also allow for legitimate disconnect time (sports, leisure activities) and sensor changes.

**Table 2:** Goals based on devices used.

Glucose monitoring device	Insulin delivery method	Glucose monitoring goal	Mealtime goal
Glucometer	MDI^a^	SMBG^b^	Enter in Glooko Mobile
Glucometer	CSII^c^	SMBG	Enter in pump
CGM^d^	MDI	CGM wear time	Enter in Glooko Mobile
CGM	CSII	CGM wear time	Enter in pump

^a^MDI: multiple daily injections.

^b^SMBG: self-monitored blood glucose.

^c^CSII: continuous subcutaneous insulin infusion.

^d^CGM: continuous glucose monitor.

The mealtime goal for participants who use a glucometer only (no CGM) is to check their glucose before they enter their carbs. Those who use MDI will enter their carb counts in the Glooko app. Participants who use CSII will enter their carb value in their pump. A glucose check must be documented within 30 minutes of the carb entry, an evidence-based criterion [56] that should occur at least 3 times per day according to clinical practice guidelines [57]. An algorithm based on the type of devices used will evaluate whether the mealtime goal was met. The daily mealtime target is 3 properly timed paired glucose levels and carb count values.

**Figure 5:**
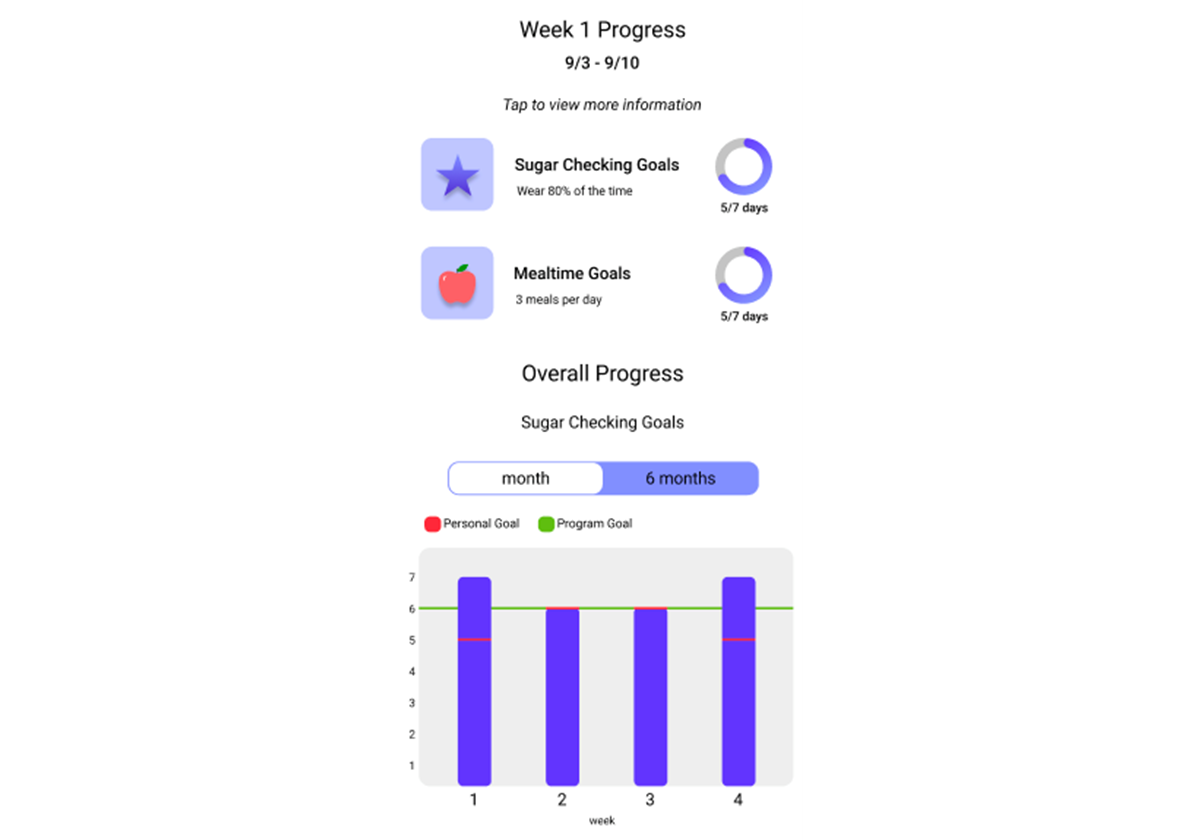
SweetGoals goal feedback graphs—continuous glucose monitor user.

The weekly goal for all participants is to meet the daily goal on 6 or more days per week. Each week, participants will also be asked to set personal goals for the next week for the number of days they think they can meet each daily goal, to help participants build motivation to gradually increase their self-management behaviors. Each week, participants will receive feedback messages and graphical reports about goals met (see Figure 5 for an example of a participant who uses a CGM). The app will send reminders to upload device data and set personal goals.

### Incentives Component

**Figure 6:**
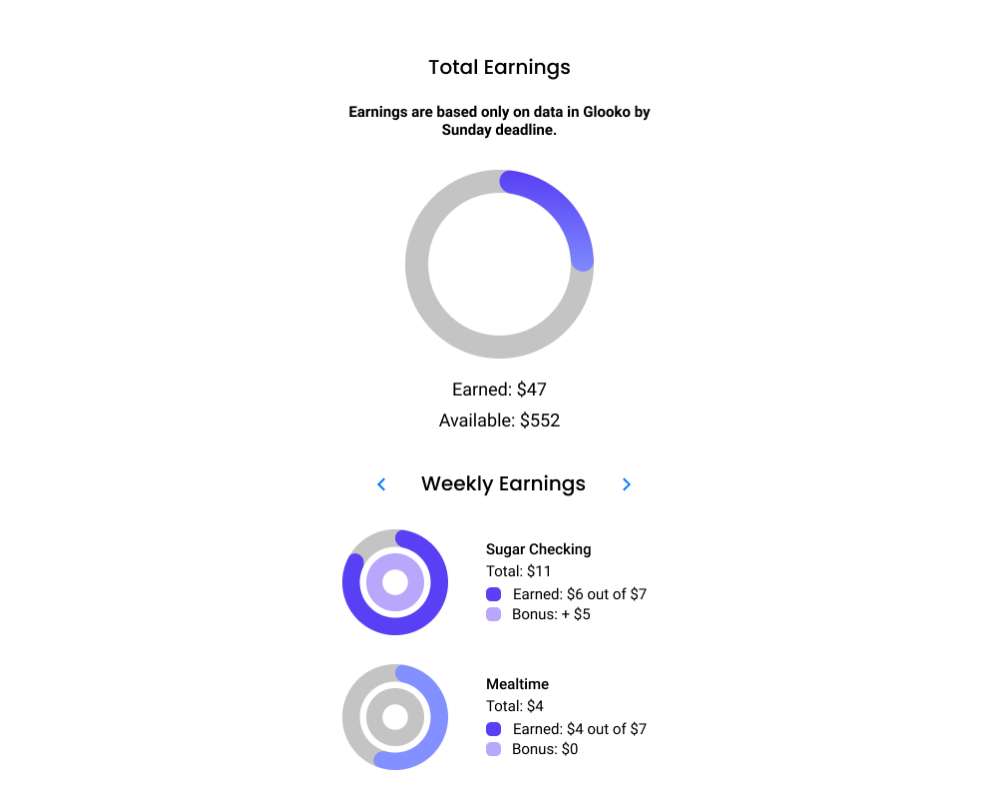
SweetGoals incentives feedback graphs.

Participants assigned to the incentive group will earn $1 per day for meeting each daily goal (glucose monitoring, mealtime behaviors) and will receive a $5 bonus for meeting the weekly target of 6 or more days per week meeting each goal, for a total of $24 maximum per week (approximately $3.50 per day). The maximum earnings for all goals are $600 across 25 weeks. The app will provide messages and graphical reports about incentives (see Figure 6). Incentives accrue weekly throughout the 25 weeks but are paid weekly from weeks 1 to 11, with payments fading in frequency from weeks 12 to 25 (ie, paid at weeks 13, 16, 20, and 25). Fading of reinforcement delivery has been shown in human and animal research to engender resistance to extinction after reinforcers end, increasing the maintenance of behavior change beyond the intervention period [58-62]. For this reason, we have incorporated a lengthy (3-month) fading period to promote maintenance of improvements in daily self-management.

This fading procedure is used to promote habit automaticity and long-lasting improvements in adherence and HbA_1c_. We have successfully used this fading paradigm in our prior studies, which resulted in no weakening of intervention effects on HbA_1c_ up to 6 months after the end of the intervention.

### Web Health Coaching Component

Participants assigned to the coaching group will receive 16 web health coaching sessions by a bachelor’s level health coach. Weekly coaching sessions occur from weeks 1 to 11, and then fade over the second half of the intervention with 4 sessions held during weeks 12 to 25 (weeks 13, 16, 20, and 25). Coaches will use their own chat interface in SweetGoals and text messages to communicate with participants. Coaches will also remind participants of meetings, offer encouragement, and respond to any messages the participant sends. In each meeting, coaches will review weekly glucose monitoring and mealtime goals and incentives earned in the context of diabetes device data reviewed using the Glooko website. Motivational exercises are completed in the first few meetings to develop rapport and allow the young adult to explore and reflect on their self-management strengths and challenges [63]. The young adult will work together with the coach to identify barriers to adherence. Coaches will also teach a structured problem-solving method to the young adult [64].

During the second half of the intervention, as coaching sessions are spaced at increasing intervals, participants are encouraged to review their diabetes data weekly on the Glooko website or app and complete the problem-solving steps on their own to address self-management concerns. Success and challenges with independent problem solving are reviewed at coaching sessions during the fading period. Throughout the intervention, if the young adults experience challenges meeting glucose monitoring or mealtime goals, coaches encourage them to use the problem-solving steps to develop a plan to improve adherence. As they gain more success meeting those goals, they are encouraged to select new goals regarding glucose levels and problem solve potential barriers to achieving those goals. Participants are always encouraged to reach out to their providers with any concerns about the insulin regimen, hypoglycemia, or hyperglycemia as coaching does not address medical management of diabetes. The structure of the problem-solving method is constant across participants; however, the content is highly personalized based on the unique challenges faced by each young adult.

Coaches receive training in communication skills consistent with a motivational interviewing approach [65]. Importantly, motivational interviewing has been identified as an effective approach for improving diabetes outcomes clinical trials [35,66,67]. Coaches are also trained in providing instruction and support in problem-solving [64,68]. Training involves didactic material and extensive role play practice.

### Diabetes Care

Participants in all conditions will receive ongoing diabetes treatment from their current medical provider. Study staff will not provide medical care or intervention. The project includes an endocrinologist who will monitor device data regularly in Glooko, and research staff will encourage participants to follow-up with their medical provider as necessary.

### Measures

Measures will be collected at baseline, 6 months (end of intervention), and 12 months unless otherwise noted. Assessments will not be blinded. All measures will be completed by participants online via the app or LimeSurvey or are objective measures (eg, device downloads, HbA_1c_ tests, or recorded directly via the app).

#### Demographic Characteristics

At baseline, demographics will be collected (eg, age, sex, race/ethnicity, insurance type). We will assess socioeconomic status using a single item measure appropriate for young adults [69]. We will assess diabetes indicators (eg, duration of diabetes, device use, past 12-month frequency of severe hypoglycemic events defined as episodes of documented or presumed low blood glucose that resulted in seizure or loss of consciousness [70] and hospitalization for diabetic ketoacidosis). Additionally, we will ask if the participant has been suspected of having a COVID-19 infection.

#### Primary Outcome

The primary outcome will be HbA_1c_ assessed using a Clinical Laboratory Improvement Amendments–waived nonfasting whole blood HbA_1c_ test (AccuBase A_1c_ test kit by DTI Laboratories) mailed to the participant (with a postpaid return envelope) at each assessment. Samples are stable at room temperature for 21 days after collection, and results are available within 48 hours of receipt. AccuBase is FDA-cleared and uses capillary tube collection. Lab testing uses high-performance liquid chromatography, including abnormal hemoglobin screening. This method has been used effectively in a large national web-based study [71].

#### Secondary Outcomes

Secondary outcomes will include key self-regulation constructs including adherence, self-efficacy, and problem solving. Adherence to glucose monitoring and mealtime behaviors will be assessed during the 30 days prior to each assessment by calculating the percentage of days meeting each goal from device data. Diabetes management self-efficacy and outcome expectations will be assessed using the 10-item Self-Efficacy for Diabetes Management scale [72] that has shown strong relations with adherence among young adults with T1D [16]. Problem-solving skills will be assessed using the 10-item version of the Social Problem-Solving Inventory–Revised (α=.85) [73,74]. To assess symptoms of diabetes distress (a moderator), the 28-item T1-Diabetes Distress scale (α=.91) [75,76] will be used. Additionally, behavioral and emotional problems will be assessed with the 34-item version of the Counseling Center Assessment of Psychology Symptoms (α=.91 with all items), with the suicidality and desire to harm others items removed [77]. Self-regulation will be assessed using a brief self-regulation scale [78], a 12-item measure. Body awareness will be measured with the Body Awareness Questionnaire (18 items; α=.82) [79]. Hypoglycemia awareness will be measured by a single item [80]. Baseline levels of technology experience will be measured using the Technology Readiness Index (16 items) [81] and a novel measure based on Venkatesh et al [82] which asks about experience with specific types of technologies. We will also assess past 30-day substance use frequency (tobacco, alcohol, cannabis, other drugs) with items adapted from the 2018 Monitoring the Future survey [83], the National Survey on Drug Use and Health [84], and the Tobacco, Alcohol, Prescription medication, and other Substance use tool [85].

#### Implementation, Satisfaction, and App Metrics

For participants who receive human coaching, satisfaction with the human health coach will be assessed at the beginning, during, and the end of the program with the Session Alliance Inventory (α=.94) [86] and an adaption of the Working Alliance Inventory [87,88]. Satisfaction with the SweetGoals digital coach will be assessed at the beginning, during, and at the end of the program with the Session Alliance Inventory [86] and (at the end of the 6-month intervention period only) an adaption of the Working Alliance Inventory [87,88]. We will assess usability of the app components with items from the Usefulness, Satisfaction, and Ease of Use questionnaire (α=.98) [89] and overall app satisfaction with items adapted from Wixom & Todd [90] 3 times (early, midway, and at the end of the 6-month intervention period). In addition, the app will collect engagement metrics including the percentage of messages from digital coaches responded to within 24 hours of receipt and the mean length of the delay between when messages are sent by the app and accessed by the participant. These data will be used to explore objective app engagement and whether satisfaction and app engagement were better when receiving incentives or coaching.

#### Health Coaching Fidelity

Coaches will complete a detailed checklist after each meeting. In addition, fidelity coding of one randomly selected session for each participant will be completed [91]. Undergraduate coders will complete an extensive training protocol in the rating system. Coders will double code a randomly selected subset (25%) of sessions, and reliability will be tracked on an ongoing basis to ensure reliability of more than 80%.

### Statistical Analysis

All subjects, once randomized, will be included in the intent-to-treat sample. We will strive to collect all primary and secondary outcomes even if a participant does not engage in assigned interventions. Data will be stored in the Research Electronic Data Capture, MobileCoach, and a secure Dartmouth server.

#### Missing Data

As in any study, missing values may occur due to dropout (anticipated to be less than 20% by the end of 12 months), inability to reach a participant for follow-up, item nonresponse, or gaps in or missing device data. The proposed structural equation modeling (SEM) and linear mixed effects models (LMMs) use all available data and are robust to outcome data that are missing at random.

#### Primary Outcome Analyses

The primary aim is to test the main effects of incentives and coaching on HbA_1c_. We will also test the interaction between components (eg, the synergistic effect of adding one component to another). Two effect-coded indicators (see Collins [51] for detailed justification for using effect coding in the analysis of data from factorial designs) will be created, one for each component: the indicator for incentives (yes vs no) will differentiate between those offered incentives (coded +1; conditions 3 and 4 in Table 1) and those who were not (coded –1; conditions 1 and 2 in Table 1); the indicator for coaching (yes vs no) will differentiate between those offered coaching (coded +1; conditions 2 and 4 in Table 1) and those who were not (coded –1; conditions 1 and 3 in Table 1). To assess the impact of each component on HbA_1c_ over time (baseline, 6 months after, and 12 months after), an LMM will be fit. The model will include fixed effects for time, two component indicators, the component × component interaction, two component × time interactions, and the component × component × time interaction as fixed effects. The LMM will also include random effects for the intercept and time to account for within-person correlation. Based on this LMM, we will test the hypothesis that the yes level of each component (vs no) results in lower HbA_1c_ over time via the component × time interaction parameters.

We calculated the power for testing effects on HbA_1c_ based on the following assumptions: (1) models will include baseline HbA_1c_; (2) a within-person correlation between baseline HbA_1c_ and HbA_1c_ at each follow-up assessment of 0.6 (based on our pilot study); and (3) a standard deviation for HbA_1c_ of 1.2, based on our pilot data. Table 3 shows detectable main effects and interactions in terms of Cohen *d* [92] and HbA_1c_ mean differences. Given n=240 to n=270 (based on 80% to 90% retention), we will be able to detect small effect sizes (Cohen *d*=0.27 to *d*=0.29) with 80% power.

**Table 3:** Aim 1 power for main effects and interaction between components.

Retention %	N	Power %	2-sided *P* value	Cohen *d*	HbA_1c_ difference %
90	270	80	.05	0.27	0.32
85	255	80	.05	0.28	0.33
80	240	80	.05	0.29	0.35

#### Secondary Outcome Analyses

Secondary analyses will evaluate change in 4 potential mechanisms of action of incentives and coaching (and their interaction) on HbA_1c_ at 6 months: glucose monitoring adherence, mealtime adherence, self-efficacy, and problem solving. We will follow the methods recently outlined for conducting mediation analysis in a factorial design with multiple mediators [93]. The mediation model will be tested using SEM with full maximum likelihood estimation, as outlined by MacKinnon et al [94]. The models will test effects of (1) each component and their interaction on the 4 mediators assessed at 6 months, (2) each mediator on HbA_1c_ at 12 months, and (3) each component and their interaction on HbA_1c_ at 12 months. Models will control for baseline levels of HbA_1c_ and the mediators. This model will evaluate the indirect effect of each component on HbA_1c_ through each mechanism, thereby testing whether the effect of coaching and incentives on HbA_1c_ is through increasing each of the 4 potential mechanisms.

#### Exploratory Analyses

Informed by the approach of Yardley et al [95], these analyses aim to determine whether app engagement and use metrics predict improvements on HbA_1c_ outcomes. We will first examine how engagement metrics differ between those receiving incentives versus no incentives and coaching versus no coaching. Use metrics will include the percentage of messages responded to within 24 hours of receipt and the mean length of the delay between when messages are sent and accessed. In LMM models predicting HbA_1c_, we will adjust for one or both components if we find significant component effects on engagement metrics. Effects of engagement metrics on HbA_1c_ outcomes are of interest in terms of future refinement and development of the SweetGoals app, suggesting critical app features that may be important to retain in future interventions or features that appear to have less impact and may need to be improved upon in future research [95].

## Results

We anticipate recruiting 300 young adults in a 36-month period (approximately 8 per month). The anticipated date of enrollment of the first participant is February 2021. We expect that data collection will be complete by February 2025. We expect to complete analyses of the primary and secondary outcomes by December 2025.

## Discussion

### Design Innovations

This study focuses on investigating the utility of two intervention components, seeking to optimize an intervention for improving glycemic outcomes among young adults with T1D. Innovations include recruiting participants with T1D into an intervention via social media and intervention delivery via an app plus coaching and incentives both delivered remotely, strategies that may be vital to reaching the targeted, understudied, and underserved population of young adults. In addition, this is the first intervention designed to target adherence via incentives among patients using diverse glucose measurement methods (glucometer, CGM) and methods of insulin delivery (MDI, CSII). This trial also makes innovative use of existing diabetes device technology by integrating a diverse array of devices with an app to automate tracking of and feedback on daily adherence habits. This digital and web-based model is designed to be broadly applicable across the range of health conditions in which patients struggle with self-management.

### Design Considerations

The study design was informed by research evidence across multiple domains. For example, the focus on young adults was based on evidence suggesting they are at unique risk for above target HbA_1c_ levels and that periods of above target HbA_1c_ levels during these years have a long-lasting negative impact on health [96]. We chose to focus on early young adulthood (ages 19 to 25 years) due to their higher risk for above target HbA_1c_ [97] and possible developmental differences across the later 20s and early 30s. Further, we chose to target young adults outside the traditional clinic setting directly via social media, empowering them to address their diabetes self-management. Many young adults are less compliant with obtaining regular medical care [98], which suggests it may be important to offer services to young adults with T1D outside the endocrinology setting to problem solve their barriers to clinical care and encourage them to schedule regular visits with their provider. This proposed model focuses on directly engaging participants rather than targeting changes in provider behavior. As such, it offers a practical outreach approach that could be deployed nationally and adopted by medical practices, health systems, or insurers outside of office visits to engage young adults with T1D in working together with their provider and managing their own health.

#### Intervention Targets

There were several decisions made in relation to our choice of particular adherence behaviors to target. Key to effectively using incentives to change behavior is the identification of specific, objectively verifiable, targets. Research on diabetes has long identified self-monitored blood glucose (SMBG) as a fundamental adherence behavior related to better glycemic control [99], and our pilot research focused on increasing SMBG. However, the increasing use of CGM (approximately 25% in the T1D Exchange sample [97]) led us to develop a strategy to include all participants with above target HbA_1c_, using any combination of diabetes devices. This choice required us to identify a key adherence behavior necessary for CGM use to have a positive impact on glycemic control. Research suggests that CGM wear time is the analog to SMBG that positively impacts glycemic control [100]. Of note, high wear time is defined as providing 80% of expected glucose values each day, based on a review of CGM studies and the recent international consensus statement [101]. By selecting targets that allow the inclusion of participants using all current glucose monitoring devices, we have greatly increased the intervention generalizability.

There are many other self-management behaviors in addition to glucose monitoring necessary to achieve below target HbA_1c_ levels, and these primarily reflect timely and accurate insulin dosing. In selecting additional specific, objectively verifiable targets related to insulin dosing we needed to address multiple challenges. Some of these include the different regimens and rules associated with use of MDI versus CSII, highly individualized insulin dosing regimens across participants, and diverse circumstances under which the dosing rules should be altered (eg, when ill, due to exercise, due to nutritional factors other than simply the number of carbs consumed). It is also not possible to objectively verify for a patient who uses MDI the timing and amount of insulin delivered. For these reasons, we opted to focus on mealtime behaviors involving a glucose value less than 30 minutes prior to an entered carb value, a pair of behaviors that are fundamental to accurate insulin dosing and that are required of all participants regardless of method of insulin dosing.

Ideally, mealtime adherence reflects 3 steps: a properly timed glucose check, entry of the number of carbs consumed, and correct insulin dosing. There are few data regarding carb counting among young adults who do not use CSII. However, research has investigated adherence among young adults using CSII [56,102-106]. Overall, adherence is highly variable across individuals, and days on which a mealtime bolus occurs more than 3 times are significantly correlated with HbA_1c_ [103,105,107,108]. Participants who complete these first two steps (glucose check and carb entry) are likely to deliver a bolus, as supported by research showing that the sequence of a properly timed glucose check and a carb entry without a bolus occurred less than 1% of the time [56].

In selecting adherence targets, we also considered targeting individual glucose levels or time in the target glucose range. Unfortunately, for participants who do not use CGM, these data are sparse and potentially not representative of daily fluctuation in glucose levels. Our selected mealtime behaviors are more actionable and are key adherence behaviors for participants using CGM, even when hybrid closed-loop systems are in widespread use [109-114]. Further, access to such systems is often allowed only for participants showing adherence and moderate HbA_1c_ elevations. For example, in the recent closed-loop study with patients with above target HbA_1c_ levels, participants with HbA_1c_>10% were excluded and participants were required to show use of CGM for at least 12 days and use of the bolus calculator for at least 75% of meal boluses over 2 weeks prior to randomization [115]. Finally, discontinuation rates for such systems may be high [116], suggesting the continued need for solutions to improve outcomes for participants using diverse treatment regimens.

#### Inclusion of Incentive Component

Some research on the use of incentives to promote health behavior related to diabetes have not shown significant benefits. For example, a scoping review [117] reported that using incentives for health behavior related to type 2 diabetes showed limited efficacy. The review highlights several critical issues related to the design of incentive interventions that can result in poor outcomes. For example, ineffective incentive programs typically offer infrequent rewards provided long after the target behavior occurs. To be effective, incentives should be provided frequently and immediately after the targeted behavior. Ineffective programs often target one-time behaviors (eg, attending a clinic visit). Instead, incentives should target learning new, daily healthy habits. Consistent with best practices, our intervention uses immediate rewards, focuses on daily health behaviors, incorporates behavioral economic principles of goal setting to engage commitment to behavior change, and provides frequent feedback.

Concern is sometimes expressed that incentives may undermine intrinsic motivation; however, the undermining effect of rewards on intrinsic motivation appears limited to simple tasks for which motivation is initially high [118]. When baseline levels of incentivized behaviors (and motivation) are low (as they generally are for health-related behaviors such as adherence among those with above target HbA_1c_), there is no evidence for a negative impact on intrinsic motivation [119]. In fact, studies have shown that incentive interventions can increase intrinsic motivation [120] and engage deliberative cognitive processes related to self-regulation to offset automatic selection of unhealthy but reinforcing behaviors [121]. Incentives can also build sustained habits [122], and habits with high automaticity may be stronger predictors of health behavior than positive intentions [123,124].

Because concern is sometimes raised about the potential for dissemination of financial incentive interventions, this study will experimentally test the impact of the incentive component of SweetGoals. In recent years, use of incentive interventions has greatly expanded across the United States. These include deploying incentives for abstinence from substance use across 94 locations in the Veterans Affairs system [125], workplace wellness programs (offered by 80% of employers with more than 1100 employees) many of which include financial incentives or penalties for employees with chronic conditions [126], offering financial incentives specific to diabetes or prediabetes (type 2 diabetes) conducted in federally qualified health centers and large health systems (Kaiser Permanente, Cleveland Clinic) [127-131], and programs offering incentives for Medicaid enrollees with prediabetes for weight loss [132] and for enrollees with hypertension or diabetes who meet health targets [133]. These efforts show the increasing dissemination of the use of financial incentives to change health behaviors in the United States and highlight the need to enhance science-based guidance to inform such programs.

### Conclusion

This study is designed to test the role of two evidence-based behavioral intervention components (incentives and health coaching) in supplementing a mobile health approach to improve glycemic outcomes among high-risk young adults with T1D who have above target HbA_1c_ levels. We will also test the effect of these intervention components on key self-regulatory mechanisms hypothesized to be impacted by these components, including two aspects of adherence (glucose monitoring and mealtime behaviors), plus self-efficacy and problem solving. Overall, results will provide critical data on enhancements to a digital intervention for T1D that are highly disseminable. They will also advance the field of theoretically driven interventions aimed at improving self-management and glycemic outcomes among high-risk young adults with elevated HbA_1c_ levels.

## Abbreviations

CGM: continuous glucose monitor
CSII: continuous subcutaneous insulin infusion
CSS: Christian Social Health Insurance Company of Switzerland
SEM: structural equation modeling
HbA_1c_: hemoglobin A_1c_
LMM: linear mixed effects model
MDI: multiple daily injections
SMBG: self-monitored blood glucose
T1D: type 1 diabetes
